# The complete mitochondrial genome of red-clawed crab *Chiromantes haematochir* (Sesarmidae: Grapsidae)

**DOI:** 10.1080/23802359.2018.1536452

**Published:** 2018-11-25

**Authors:** Qingyue Li, Caoling Xu, Chong Wang, Gang Liu

**Affiliations:** School of Life Sciences, Anhui Medical University, Hefei, P. R. China

**Keywords:** *Chiromantes haematocheir*, complete mitochondrialgenome sequences, phylogenetic tree

## Abstract

The complete mitogenome of *Chiromantes haematocheir* is 15 899 bp in length contains the typical set of 37 genes, including 13 protein-coding genes (PCGs) (*ATP6*, *ATP8*, *COI-III*, *ND1-6*, *ND4L*, and *Cyt b*), two rRNAs (12S rRNA and 16S rRNA), 22 tRNAs, and a putative CR (D-loop). All PCGs start with an ATG codon except ND1 start with ATT and ND3 start with ATA. TAA is the most frequent stop codon, although COI end with TA-, and COII stop with the single nucleotide T-. The new mtDNA sequence contains 12S rRNA and 16S rRNA of rRNAs, separated with tRNA^val^. All tRNAs possess the typical clover leaf secondary structure except for tRNA^Ser(AGN)^ and tRNA^Leu(CUN)^, which lacks a dihydroxyuridine (DHU) arm. The CR is 826 bp in length, located between 12S rRNA and tRNA^Glx^. The phylogenetic trees support *Chiromantes haematochir* has close relative with *Sesarmops sinensis* and *Sesarmane glectum*.

*Chiromantes haematocheir* is known under the common name red-clawed crab, is a mudflat crab of the family Sesarmidae (subfamily Sesarminae), which is endemic to East Asia. It is quite distinct from the other species placed in the genus Chiromantes, and the genus may be restricted to this one species (Peter et al. 2008). The sample of *Chiromantes haematocheir* was collected from Fushun City (123°55′07.15″ E, 41°52′42.23″ N), Liaoning Province in May, 2018. The sample was stored in the Biology Specimen Room of the College of Life Sciences, Anhui Medical University, China (Sample code is AMHU-SSW20180502). The DNA sample was stored at −80 °C in the Cancer Cell Biology Laboratory, College of Life Sciences, Anhui Medical University. The complete mtDNA sequence of *Chiromantes haematocheir* has been assigned GenBank accession number MH457175.

The length of the complete mtDNA sequence is 15 899 bp, is similar to other *Chiromantes* species (Xing et al. 2016). It contains the typical set of 37 genes, including 13 protein-coding genes (PCGs) (*ATP6*, *ATP8*, *COI-III*, *ND1-6*, *ND4L*, and *Cyt b*), two rRNAs (12S rRNA and 16S rRNA), 22 tRNAs, and a putative CR (D-loop). The overall base composition for the mtDNA sequence is as follows: A 37.6%, C 15.0%, G 9.1%, and T 38.3%. The new sequence has higher A + T content (75.9%) and lower C + G content (24.1%), similar to other invertebrate species (Xing et al. 2016). Through the 13 PCGs, the longest one is ND5 (1728 bp), and the shortest is ATP8 (159 bp), similar with the other species (Xiao et al. 2012). All PCGs start with an ATG codon except ND1 start with ATT and ND3 start with ATA. TAA is the most frequent stop codon, although COI end with TA–, and COII stop with the single nucleotide T–. The new mtDNA sequence contains 12S rRNA and 16S rRNA of rRNAs, which are located between tRNA^Phe^ and D-loop, separated by tRNA^Val^. The 12S rRNA is 826 bp long and the 16S rRNA is 1343 bp in length. All tRNA genes possess the typical clover leaf secondary structure except for tRNA^Ser(AGN)^ and tRNA^Leu(CUN)^, which lacks a dihydroxyuridine (DHU) arm. The non-coding regionsinclude a control region (D-loop) and a few intergenic spacers. The D-loop is located between 12S rRNA and tRNA^Glx^, and is 826 bp in length.

Phylogenetic trees were estimated using ML and BI methods, based on the complete mtDNA of seven Sesarmidae species, and corresponding *Coreana raphaelis* (NC_007976) sequence was used as an outgroup, sharing similar topologies and high node support values (Figure 1). The results indicated *Chiromantes haematochir* has close relative with *Sesarmops sinensis* and *Sesarmane glectum*.

**Figure 1.**
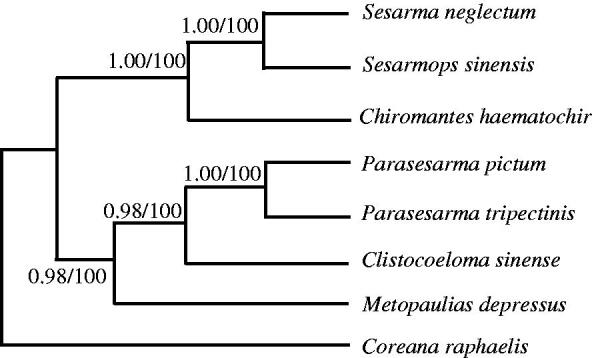
Phylogenetic relationships among the seven Sesarmidae species based on complete mtDNA sequences. Numbers at each node are Bayesian posterior probabilities (left) and maximum likelihood bootstrap proportions (estimated from 100 pseudoreplicates) (right). The accession number in GenBank of seven Sesarmidae in this study: *Sesarmops sinensis* (NC_031851), *S. neglectum* (NC_031851), *Chiromantes haematochir* (MH457175), *Parasesarma tripectinis* (NC_030046), *P. pictum* (MG580780), *Metopaulias depressus* (NC_030535), and *Coreana raphaelis* (NC_007976).

## Nucleotide sequence accession number

The complete mtDNA sequence of *Chiromantes haematochir* has been assigned with GenBank accession number MH457175.
